# Supplementary material for: Bugs That Can Resist Antibiotics but Not Men: Gender-Specific Differences in Notified Infections and Colonisations in Germany, 2010–2019
Source: Microorganisms. 2021 Apr 22;9(5):894. doi: 10.3390/microorganisms9050894 (PMC8143559; doi:10.3390/microorganisms9050894)
Supplement: Supplementary file 1 [file microorganisms-09-00894-s001.zip › microorganisms-1163649-supplementary.pdf]

Table S1. Results of univariable and multivariable analysis using Poisson regression for notified invasive infections with Methicillin-resistant *S. aureus* (MRSA), 2010-2019, and infections or colonisations with carbapenem-non-susceptible *Acinetobacter spp.* (CRA) and Enterobacterales (CRE), 2017-2019

		**MRSA (n = 34,551)**		**CRA (n = 2,278)**		**CRE (n = 12,055)**	
**Variable**	**Value**	**Univariable analysis** Crude IRR (95%CI)	**Multivariable analysis** Adj. IRR* (95%CI)	**Univariable analysis** Crude IRR (95%CI)	**Multivariable analysis** Adj. IRR* (95%CI)	**Univariable analysis** Crude IRR (95%CI)	**Multivariable analysis** Adj. IRR* (95%CI)
**Gender**	Female	ref.	ref.	ref.	ref.	ref.	ref.
	Male	1.8 (1.7-1.8)	2.3 (2.2-2.3)	1.9 (1.8-2.1)	2.2 (2.0-2.4)	1.6 (1.6-1.7)	1.9 (1.8-2.0)
**Age group**	< 1	13 (10-17)	13 (10-17)	4.7 (2.5-8.8)	4.6 (2.5-8.7)	15 (13-18)	15 (13-18)
	1 – 9	ref.	ref.	ref.	ref.	ref.	ref.
	10 – 19	0.82 (0.63-1.1)	0.81 (0.62-1.1)	1.3 (0.78-2.0)	1.3 (0.79-2.1)	0.67 (0.55-0.83)	0.68 (0.55-0.84)
	20 – 29	1.9 (1.5-2.4)	1.9 (1.5-2.3)	2.9 (1.9-4.4)	2.9 (1.9-4.3)	1.5 (1.3-1.8)	1.5 (1.2-1.7)
	30 – 39	3.2 (2.6-3.9)	3.2 (2.6-3.9)	2.9 (1.9-4.4)	2.8 (1.9-4.3)	1.7 (1.4-2.0)	1.7 (1.4-2.0)
	40 – 49	6.6 (5.4-8.0)	6.4 (5.3-7.8)	3.7 (2.5-5.5)	3.7 (2.5-5.5)	2.1 (1.8-2.5)	2.1 (1.8-2.5)
	50 – 59	17 (14-20)	17 (14-20)	6.2 (4.2-9.1)	6.4 (4.3-9.4)	4.0 (3.4-4.6)	4.0 (3.5-4.7)
	60 – 69	41 (34-49)	42 (34-50)	12 (8.0-17)	13 (8.6-18)	8.6 (7.4-9.9)	9.0 (7.8-10)
	70 – 79	87 (72-100)	88 (73-110)	20 (14-29)	22 (15-32)	14 (12-17)	15 (13-18)
	80 +	120 (100-150)	140 (120-170)	14 (9.6-21)	16 (11-24)	14 (12-16)	16 (13-18)
**Federal state of residence**	Baden-Württemberg	1.1 (0.93-1.2)	1.4 (1.2-1.6)	2.1 (1.2-3.6)	2.8 (1.6-4.8)	2.2 (1.7-2.7)	2.8 (2.2-3.5)
	Bavaria	1.3 (1.2-1.5)	1.8 (1.6-2.0)	2.5 (1.4-4.2)	3.2 (1.9-5.5)	2.2 (1.8-2.7)	2.8 (2.2-3.5)
	Berlin	4.2 (3.7-4.8)	5.9 (5.2-6.8)	8.2 (4.8-14)	11 (6.5-19)	4.9 (4.0-6.1)	6.7 (5.3-8.3)
	Brandenburg	3.1 (2.7-3.6)	3.6 (3.2-4.1)	2.1 (1.1-3.8)	2.4 (1.3-4.4)	1.9 (1.5-2.4)	2.1 (1.7-2.7)

	Bremen	ref.	ref.	ref.	ref.	ref.	ref.
	Hamburg	1.5 (1.3-1.7)	2.1 (1.8-2.5)	7.1 (4.1-12)	10 (5.8-17)	3.7 (3.0-4.7)	5.2 (4.1-6.5)
	Hessen	1.9 (1.7-2.1)	2.5 (2.2-2.8)	5.4 (3.2-9.3)	7.1 (4.1-12)	4.7 (3.8-5.8)	6.0 (4.8-7.4)
	Lower Saxony	3.5 (3.0-3.9)	4.3 (3.8-4.9)	1.9 (1.1-3.3)	2.4 (1.4-4.1)	1.4 (1.1-1.7)	1.7 (1.4-2.2)
	Mecklenburg-Vorpommern	4.3 (3.7-4.9)	5.0 (4.4-5.7)	1.3 (0.63-2.5)	1.5 (0.74-3.0)	1.2 (0.93-1.6)	1.4 (1.1-1.9)
	North Rhine-Westphalia	3.7 (3.3-4.2)	4.8 (4.2-5.4)	3.7 (2.2-6.3)	4.8 (2.8-8.1)	2.9 (2.3-3.5)	3.6 (2.9-4.5)
	Rhineland-Palatinate	1.6 (1.4-1.9)	2.1 (1.8-2.4)	2.2 (1.2-3.9)	2.7 (1.5-4.8)	2.6 (2.1-3.2)	3.1 (2.5-3.9)
	Saarland	1.8 (1.5-2.1)	2.1 (1.8-2.5)	0.80 (0.32-2.0)	0.96 (0.39-2.4)	2.3 (1.7-3.0)	2.6 (2.0-3.4)
	Saxony	3.5 (3.1-4.0)	3.9 (3.4-4.4)	2.6 (1.5-4.6)	3.0 (1.7-5.3)	2.6 (2.1-3.3)	2.9 (2.3-3.7)
	Saxony-Anhalt	4.3 (3.8-4.9)	4.8 (4.2-5.4)	1.6 (0.84-3.0)	1.8 (0.96-3.4)	3.2 (2.5-4.0)	3.5 (2.8-4.5)
	Schleswig-Holstein	2.8 (2.4-3.2)	3.4 (3.0-3.9)	1.9 (1.0-3.4)	2.3 (1.3-4.2)	1.7 (1.3-2.1)	2.0 (1.6-2.5)
	Thuringia	2.7 (2.3-3.1)	3.0 (2.6-3.5)	2.4 (1.4-4.5)	2.9 (1.6-5.2)	2.9 (2.3-3.7)	3.3 (2.6-4.1)
**Year of notification**	2010	ref.	ref.				
	2011	1.1 (1.1-1.2)	1.1 (1.1-1.2)				
	2012	1.2 (1.2-1.3)	1.2 (1.1-1.2)				
	2013	1.2 (1.1-1.2)	1.1 (1.1-1.2)				
	2014	1.0 (0.99-1.1)	0.98 (0.94-1.0)				
	2015	0.96 (0.91-1.0)	0.91 (0.87-0.95)				
	2016	0.84 (0.80-0.88)	0.79 (0.76-0.83)				
	2017	0.74 (0.71-0.78)	0.70 (0.66-0.73)	ref.	ref.	ref.	ref.
	2018	0.64 (0.61-0.67)	0.59 (0.56-0.62)	0.99 (0.90-1.1)	0.99 (0.81-1.1)	1.1 (1.1-1.2)	1.1 (1.1-1.2)
	2019	0.47 (0.45-0.50)	0.43 (0.40-0.46)	0.90 (0.81-1.0)	0.90 (0.81-0.99)	1.4 (1.3-1.4)	1.3 (1.3-1.4)

CI: confidence interval, CRA: carbapenem-non-susceptible *Acinetobacter spp*., CRE: Carbapenem-non-susceptible Enterobacterales, IRR: incidence rate ratio, MRSA: Methicillin-resistant *S. aureus*

* adjusted for age group, federal state of residence and year of notification

Table S2: Results of age-stratified univariable analysis of infection/colonisation of men compared to women in the German population (pop.) vs the German hospitalised population (hosp.) of 2018

**Age group**	**MRSA**		**CRA**		**CRE**	
	Pop. IRR (95%CI)	Hosp. IRR (95%CI)	Pop. IRR (95%CI)	Hosp. IRR (95%CI)	Pop. IRR (95%CI)	Hosp. IRR (95%CI)
< 1	1.2 (0.88-1.6)	1.2 (0.84-1.6)	1.9 (0.64-5.5)	1.8 (0.61-5.3)	1.1 (0.87-1.3)	1.0 (0.85-1.3)
1 – 9	1.2 (0.81-1.7)	0.93 (0.64-1.4)	0.60 (0.28-1.3)	0.48 (0.22-1.0)	0.94 (0.71-1.2)	0.74 (0.56-0.98)
10 – 19	1.3 (0.88-1.9)	1.5 (1.0-2.2)	1.2 (0.66-2.3)	1.5 (0.78-2.7)	1.1 (0.77-1.5)	1.3 (0.91-1.7)
20 – 29	1.6 (1.3-2.0)	1.6 (1.2-2.0)	2.8 (1.8-4.2)	2.8 (1.8-4.2)	1.4 (1.1-1.6)	1.4 (1.1-1.6)
30 – 39	1.3 (1.1-1.5)	1.2 (1.0-1.4)	1.9 (1.3-2.7)	1.8 (1.2-2.6)	1.4 (1.2-1.7)	1.4 (1.1-1.6)
40 – 49	1.7 (1.5-1.8)	1.6 (1.4-1.8)	1.6 (1.2-2.2)	1.6 (1.1-2.2)	1.5 (1.3-1.8)	1.5 (1.3-1.7)
50 – 59	2.1 (1.9-2.2)	1.7 (1.6-1.9)	2.4 (1.9-3.1)	2.1 (1.6-2.6)	1.7 (1.6-1.9)	1.5 (1.3-1.6)
60 – 69	2.4 (2.3-2.5)	1.8 (1.8-1.9)	2.2 (1.8-2.7)	1.7 (1.4-2.0)	2.2 (2.0-2.4)	1.7 (1.6-1.8)
70 – 79	2.2 (2.1-2.3)	1.8 (1.7-1.9)	2.4 (2.1-2.9)	2.0 (1.7-2.3)	2.0 (1.9-2.2)	1.6 (1.5-1.7)
80 +	2.3 (2.2-2.4)	1.9 (1.8-2.0)	1.8 (1.4-2.2)	1.6 (1.3-2.0)	2.0 (1.8-2.2)	1.8 (1.6-1.9)
**Overall***	2.2 (2.2-2.2)	1.8 (1.8-1.8)	2.1 (1.9-2.3)	1.8 (1.7-2.0)	1.8 (1.8-1.9)	1.6 (1.5-1.6)

CI: Confidence interval, CRA: Carbapenem-non-susceptible *Acinetobacter spp.*, CRE: Carbapenem-non-susceptible Enterobacterales, IRR: incidence rate ratio, MRSA: Methicillin-resistant *S. aureus*

* Pooled Mantel-Haenszel estimates using weights proportional to the number of individuals in each stratum

Table S3. Incidence rate ratios (IRR) from univariable analysis of infection/colonisation with Methicillin-resistant *S. aureus* (MRSA), *Acinetobacter spp.* (CRA) and Enterobacterales (CRE) in 20 districts with highest pig density vs 381 remaining German districts, 20 most rural vs. 20 most urban districts and 20 highest vs. 20 least deprived districts (according to the German Index of Social Deprivation, GISD).

**Districts (n)**	**MRSA**	**CRA**	**CRE**
	IRR (95%CI)	IRR (95%CI)	IRR (95%CI)
Highest pig density (20)	1.8 (1.7-2.0)	1.9 (1.2-3.1)	1.4 (1.2-1.7)
Other districts (381)	1.8 (1.8-1.9)	2.0 (1.8-2.2)	1.7 (1.6-1.8)
Most rural (20)	2.0 (1.8-2.2)	1.1 (0.6-2.1)	2.3 (1.7-3.0)
Most urban (20)	1.7 (1.7-1.8)	2.2 (1.9-2.5)	1.7 (1.6-1.8)
High deprivation (20)	1.9 (1.7-2.0)	1.3 (0.8-2.0)	1.8 (1.5-2.2)
Low deprivation (20)	1.9 (1.7-2.1)	2.1 (1.7-2.7)	1.7 (1.6-1.9)

CI: Confidence interval, CRA: Carbapenem-non-susceptible *Acinetobacter spp.*, CRE: Carbapenem-non-susceptible Enterobacterales, IRR: incidence rate ratio, MRSA: Methicillin-resistant *S. aureus*
